# Analysis of oral bacterial communities: comparison of HOMI*NGS* with a tree-based approach implemented in QIIME

**DOI:** 10.1080/20002297.2019.1586413

**Published:** 2019-04-01

**Authors:** Robert J. Palmer, Sean L. Cotton, Alexis S. Kokaras, Pamela Gardner, Margaret Grisius, Eileen Pelayo, Blake Warner, Bruce J. Paster, Ilias Alevizos

**Affiliations:** a Oral Immunity and Inflammation Unit, National Institute of Dental and Craniofacial Research, National Institutes of Health, Bethesda, MD, USA; b Department of Microbiology, The Forsyth Institute, Cambridge, MA, USA; c Sjögren’s Syndrome Clinic, National Institute of Dental and Craniofacial Research, National Institutes of Health, Bethesda, MD, USA; d AAV Biology Section, National Institute of Dental and Craniofacial Research, National Institutes of Health, Bethesda, MD, USA; e Salivary Gland Dysfunction Unit, National Institute of Dental and Craniofacial Research, National Institutes of Health, Bethesda, MD, USA

**Keywords:** HOMI*NGS*, QIIME, supragingival dental plaque, primary Sjögren’s Syndrome, microbiome

## Abstract

**Background: **Molecular taxonomic assignments in oral microbial communities have been made using probe-matching approaches, but never compared to those obtained by more readily accepted tree-based approaches. **Objective: ** To compare community composition profiles obtained from a probe-matching approach (HOMI*NGS*) to those from a closed-ended tree-based approach (QIIME using the eHOMD database). **Design: ** HOMI*NGS *and QIIME were used for parallel analysis of ten mock community samples, and of 119 supragingival plaque samples from ecologically unique sites (sound tooth surfaces in healthy subjects, sound tooth surfaces in patients with primary Sjögren’s Syndrome, and carious lesions in Sjögren’s Syndrome patients). Linear discriminant analysis Effective Size (LEfSe) was used to identify discriminating taxa among the natural plaque samples. **Results:** Community composition profiles of all samples were congruent between the two analysis aproaches. Alpha and beta diversity of the natural plaque communities were likewise similar. Communities from pSS patients and those from individuals with normal salivary flow differed in alpha and beta diversity. Both classification approaches yielded differences in composition predicted for samples from these subject cohorts, and discriminating taxa were similar between approaches. **Conclusions:** A direct comparison demonstrates that HOMI*NGS* is largely equivalent to the tree-based approach as implemented here.

## Introduction

The oral bacterial community has been investigated for centuries because it is amenable to non-invasive sampling and because its members demonstrate broad physiological diversity that is directly relevant to human health; the microbial biofilms associated with caries and with periodontitis are recognized as unique communities with their own special ecological characteristics [1,2]. Community composition at various locations within the oral cavity has been repeatedly catalogued using cultural [3,4] and molecular [5,6] methods. Many taxa are unique to the oropharynx and respiratory tract, community composition (especially at the genus level) is remarkably consistent, and differences in composition are usually those of relative amounts rather than presence/absence of taxa [7].

The earliest molecular approach to oral community analysis was a genomic DNA binding assay called checkerboard DNA-DNA hybridization in which DNA extracted from a community was fixed on a membrane then hybridized to a set of digoxigenin-conjugated whole-genome probes prepared from single bacterial isolates [8]. This approach was superseded by reverse-capture checkerboard hybridization, *i.e*. oligonucleotide probes designed from 16S rDNA gene sequences were fixed to a membrane in a slot-blot apparatus, after which digoxigenin-labeled near-full-length 16S amplicons from the community were hybridized to the probes [9]. Many of the probes were species-specific. At that time, labor- and resource-intensive cloning studies remained the gold-standard for detailed analyses, but the semi-quantitative probe-based methodology was a practical solution and appeared frequently in the literature, *e.g*. [10–12]. The final iteration of the reverse-capture approach was a microarray (the Human Oral Microbe Identification Microarray) [13] on which roughly 300 probes referenced to a highly curated database of oral bacterial 16S rRNA gene sequences (the Human Oral Microbiome Database; HOMD) [14] were spotted onto a glass slide: hybridization of the fluorescently labeled near-full-length amplicons was assessed by imaging. More recently, the probe-based approach moved to high-throughput sequence analysis; a search program called ProbeSeq was developed to directly match the probe sequences to MiSeq V3-V4 amplicon libraries and thereby generate a semi-quantitative dataset. This high-throughput approach was called Human Oral Microbe Identification using *Next Generation Sequencing* (HOMI*NGS*). Although other approaches based on amplicon regions of better specificity for particular genera (e.g. V1-V2) became available concurrently [15], the V3-V4 HOMI*NGS* approach has been used extensively and the V3-V4 region remains in use [16]. While the probe-based approach cannot assess new taxa, it is important to note that HOMI*NGS* probes cover many uncultivated taxa including those in the genera *Treponema* and *Prevotella*, and also those in the TM7 group.

HOMI*NGS* has been shown to support and expand results of HOMIM [17], but direct comparison of HOMI*NGS* with the more generally accepted tree-based methods is lacking even though the initiatory MiSeq library lends itself to parallel analysis. In the present study, 10 mock-community mixtures of 16S rRNA gene amplicons, together with 119 MiSeq libraries prepared from supragingival plaque samples acquired from primary Sjögren’s Syndrome (pSS) patients and from subjects with normal salivary flow, are analyzed in parallel using HOMI*NGS* and a tree-based approach implemented in the QIIME pipeline.

## Materials and methods

### Study approval

All studies were carried out in accordance with approved National Institute of Health (NIH) guidelines conforming to the standards indicated by the Declaration of Helsinki. All study participants provided informed consent prior to the initiation of any study procedures. Human samples were obtained from NIH Institutional Review Board approved protocols (ClinicalTrials.gov Identifier: NCT00001196m NCT01425892, and NCT00001390) in the Sjögren’s Syndrome Clinic at the National Institute of Dental and Craniofacial Research (NIDCR) at the NIH in Bethesda, MD.

### Patient population and sampling procedures

Subjects diagnosed with primary Sjögren’s Syndrome (pSS) [18] were recruited through NIDCR Sjögren’s Syndrome Characterization (15-D-0051) and Pathogenesis (11-D-0172) protocols Healthy volunteers were recruited under the protocol Salivary Evaluation in Healthy Volunteers (94-D-0018). Healthy subjects were verified to have normal salivary flow (Table 1) and to have no active caries. Plaque was collected with a Gracey curette from the buccal surfaces of teeth located nearest to the parotid duct: #2, #3, #14 and #15. Lingual surfaces were occasionally sampled (in five of the 21 pSS subjects and in two of the 10 healthy subjects) when no enamel was present on a buccal surface due to existing restorations. Three pSS subjects had active caries, from whom nine cavitated lesions were sampled. When target teeth were missing, the nearest tooth in that quadrant was sampled. In two of the subjects with active caries, sampling of at least one corresponding mandibular tooth was required because no non-restored surfaces were present in the maxillary arch quadrant. At least one mandibular sample was likewise required for four additional pSS subjects. Mandibular sampling was not required for the healthy subjects. Four plaque samples were typically obtained from each subject, but fewer were obtained from four of the pSS subjects due to tooth loss, multiple restorations, or discomfort. Additional samples were sometimes obtained to expand sampling in individuals with carious sites.

**Table 1.** Salivary flow rates (mL/min) for subjects from which plaque samples were obtained.

	pSS			Healthy			
	Range	Mean (SD)	Median	Range	Mean (SD)		Median
# Subjects, sex	21, all f	–	–	10, 6m/4f	–		–
Age	39–74	58.7 (2.3)	59	20–52	35.3 (3.9)		32
Unstimulated flow, parotid	0–0.26	0.02 (0.01)	0	0–0.22	0.06 (0.03)		0
Stimulated flow, parotid	0–0.91	0.10 (0.05)	0	0–1.12	0.52 (0.12)		0.58
Unstimulated flow, submandibular	0–0.09	0.02 (0.01)	0	0.01–0.37	0.12 (0.04)		0.06
Stimulated flow, submandibular	0–0.48	0.08 (0.03)	0.02	0.16–1.20	0.38 (0.10)		0.28
Unstimulated total flow (drool)	0–0.59	0.08 (0.03)	0	0.15–0.61	0.33 (0.15)		0.30

### Sequence library preparation and HOMINGS analysis

Plaque was transferred to 150 µL of Tris/EDTA (TE buffer) on ice and processed within 2 h. Samples were occasionally stored at −20°C for no more than 24 h prior to processing. One µL of Epicentre Ready-Lyse lysozyme (Lucigen, Middleton USA) was added to the samples, after which they were incubated at 37°C overnight. Extraction of total nucleic acid was performed using the Epicentre MasterPure Complete DNA and RNA Purification kit (MC85200, Lucigen, Middleton USA). One hundred fifty µL 2x T&C lysis solution were added to each sample, after which 1-µL Proteinase K solution (Qiagen, Germantown USA) was added and the samples incubated at 65°C for 30 min with vortexing at 5-min intervals. Samples were placed on ice for 5 min, after which 175 µL chilled MPC protein precipitation reagent were added followed immediately by 10 sec of strong vortexing. Precipitated protein was pelleted by centrifugation at 10K x g for 10 min at 4°C. The supernatants were collected, 500 µL chilled isopropanol were added, and the tube inverted 20 times. After 10 min on ice, the samples were centrifuged again, the supernatants discarded, and the pellet washed twice with 500 µL 75% ethanol. After removal of the second ethanol supernatant, the pellets were allowed to dry at room temperature, dissolved in 25 µL TE buffer, frozen, then shipped on dry ice to the Forsyth Institute (Cambridge MA) for library preparation and HOMI*NGS* analysis.

HOMI*NGS* analysis [19] proceeds by the following steps: primary amplification of the 16S rRNA gene using general primers (forward primer NF1: CCA GRG TTY GAT YMT GGC and reverse primer 1541R: GAA GGA GGT GWT CCA DCC, and a reamplification using V3-V4 primers:

341F: AAT GAT ACG GCG ACC ACC GAG ATC TAC ACT ATG GTA ATT GTC CTA CGG GAG GCA GCAG

806R: CAA GCA GAA GAC GGC ATA CGA GAT NNN NNN NNN NNN AGT CAG TCA GCC GGA CTA CHV GGG TWT CTA AT

Amplicons were sequenced on the MiSeq platform under the following conditions: SBS chemistry, multiplexed libraries spiked with 20% Phix, 250 bp (500 cycles) paired-end (MiSeq Reagent Kit v2) to yield 441 bp/sequence. Sequences were filtered to Phred score of 25, and then the custom-written program ProbeSeq developed by Sean Cotton was used to match amplicons against a collection of 647 probes that are partial (17–40 bases) 16s rRNA sequences representative of oral bacteria at the species- or genus-level. The program matches amplicon sequences first to a set of species-specific probes, after which unmatched sequences are matched against a set of genus-level probes. The genus-level probes capture sequences of those organisms for which a species-specific probe cannot be designed. A particular genus-level probe can also match with certain species-level sequences, *i.e*. potential exists for over-representation of those organisms. However, removal of sequences that match the species-level probes prior to matching against the genus-level probes theoretically reduces overlap. Generally, 15–25% of reads within any sample match neither species – nor genus-level probes and are referred to as unassigned reads. All matched-probe abundance values were summed within each sample, and then probes with an abundance <0.01% of the summed value were removed. For results presented at the genus-level, abundances of the species-level probes were summed after which genus-level probe abundances were added to the corresponding species abundances.

### QIIME analysis

Assessment of the read quality was performed using MultiQC [20]. One hundred thirty-four FASTQ files (56% of the sample files) had a minimum Phred quality score of 25, fifty-four (23%) had minimum of score 20, and the remaining 50 (21%) had a minimum score of 18. The average score was 33.4. Base-calling quality degraded beyond 150 bp but with significant variance among samples in accordance with ranges noted earlier. For the subsequent quantitative analysis, the Phred score was set to 25 as the cutoff.

Raw Illumina FASTQ files were first demultiplexed using a custom Python script, and then quality filtered and quantitively analyzed using QIIME 1.9 [21] on Nephele (https://nephele.niaid.nih.gov). Specifically, paired-end reads were first joined using the QIIME invocation of fastq-join. Sequences with any degenerate bases (*e.g*. N), the Phred quality score less than 25 per base, and more than three consecutive low-quality base calls were filtered. Quality trimming resulted in approximately 8 million high-quality reads for 119 samples with a median of 58,000 reads per sample. All samples were included in the downstream quantitative analyses. Sequences were taxonomically classified using the expanded HOMD reference database, release 15.1 [22]. Sequences were binned into OTUs and taxonomically assigned at 99% identity using the QIIME closed-reference OTU picking workflow with SortMeRNA [23]. In the closed reference OTU picking workflow, reads were clustered against the HOMD database and any reads that did not hit a reference sequence were excluded from all downstream analyses. SortMeRNA performs sensitive, high-quality local alignment of rRNA reads against reference sequences [23]. Rare OTUs or OTUs with only one read were removed. The resulting OTU table was normalized to 20,640 reads per sample for the downstream diversity and quantitative analyses. For the comparison between the QIIME and HOMI*NGS* approaches, the HOMI*NGS* OTU table was converted into the BIOM [24] format as an input to the QIIME core diversity analysis workflow.

Diversity was assessed by calculating the Shannon diversity metric, the chao1 estimate of diversity, and the number of observed species for each sample at various sequencing depths. Specifically, the QIIME OTU table was randomly subsampled 10 times from 100 to 20,640 reads per sample in steps of 2,064 reads, and the HOMI*NGS* from 100 to 21,009 in steps of 2,100. Beta diversity estimates were calculated using weighted and unweighted UniFrac distances [25] between samples, with even subsampling at 20,640 sequences per sample for QIIME and 21,009 for HOMI*NGS* with 1,000 Monte Carlo iterations. Procrustes analysis was performed with identical parameters on both the QIIME and HOMI*NGS* OTU tables using the UniFrac distance matrices with 1,000 Monte Carlo randomizations to compute goodness of fit and visualized using weighted and unweighted PCoA. For the QIIME OTU table, phylogenetic trees were constructed using FastTree with the double precision floating-point option [26]. Trees were constructed with a set of sequences representative of the OTUs. For the HOMI*NGS* OTU table, the phylogenetic tree of the HOMD database was used. Nonparametric *t*-tests using Monte Carlo permutations to calculate the Bonferonni corrected *p* value determined the significant differences in diversity between different groups. Bar charts were also constructed to visualize the taxa present in each sample and across sample groups. Kruskal–Wallis nonparametric ANOVA tests on the OTU table identified significant changes in the relative abundance of individual OTUs between groups. LEfSe (linear discriminant analysis effective size) [27] was used to identify biomarkers between different groups using relative abundance with LDA score cutoff ≥3.

### Comparison of QIIME and probeseq using mixtures of PCR products from defined 16S sequences

Mixtures were created using differing amounts of PCR products amplified from 16S rRNA gene sequences existing as clones at The Forsyth Institute. The mixtures, consisting of 6 to 23 unique PCR products, were designed to contain sequences from organisms across a wide range of abundances in the oral microbiome. Some mixtures included sequences representative of rare organisms (*e.g. Bifiobacterium animalis* and *Mycoplasma salivarium*) in small amounts, and some included mixtures of related organisms (*e.g*. streptococci). This experiment was designed solely to compare relative abundances as determined by HOMI*NGS* and a tree-based approach for an ideal set of known sequences, not to assess quantitation accuracy for any particular sequence. Therefore, the absolute amount of any sequence in a mixture was not determined.

## Results

### Comparison of taxonomic assignments for mixtures of defined PCR products

**Figure 1.**
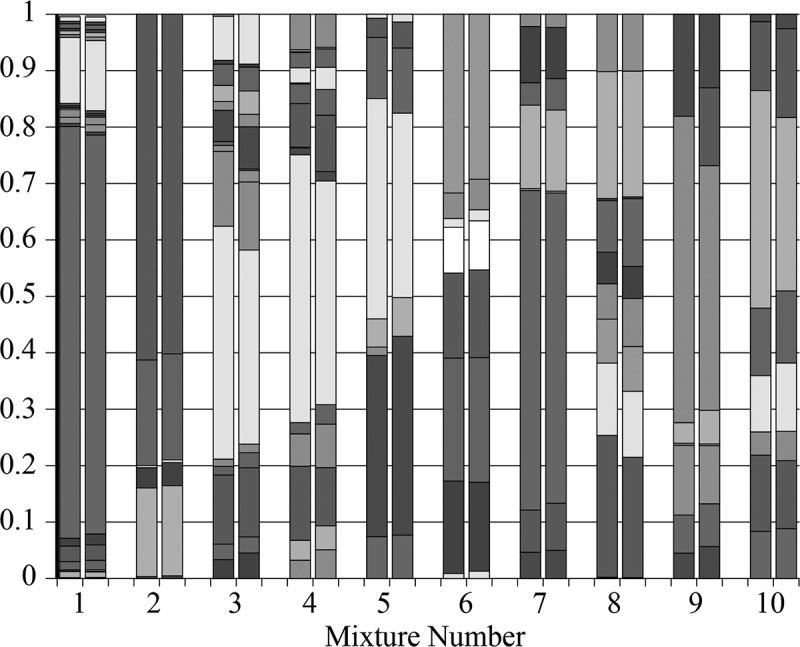
Proportional composition of 10 PCR product mixtures analyzed by QIIME (left column) and by ProbeSeq (right column). Colors represent individual sequences but are not the same for the different mixtures; 79 different products appear in the graph. Some sections in mixtures 1, 4 and 8 cannot be discerned because the amount of a particular sequence amount is low. See Supplementary Table 1 for all values.

QIIME and ProbeSeq were compared using 10 mixtures of PCR products from 16S rRNA gene sequences. Figure 1 and Supplementary Table 1 show that the analysis approaches gave congruent results. In only one case (Mixture 9) was a major difference seen: a sequence identified by ProbeSeq but not by QIIME. Importantly, variation between QIIME and ProbeSeq was low even for sequences present in the mixture at amounts less than 1% of the mixture.

### Characteristics of HOMINGS and QIIME datasets from plaque

**Figure 2.**
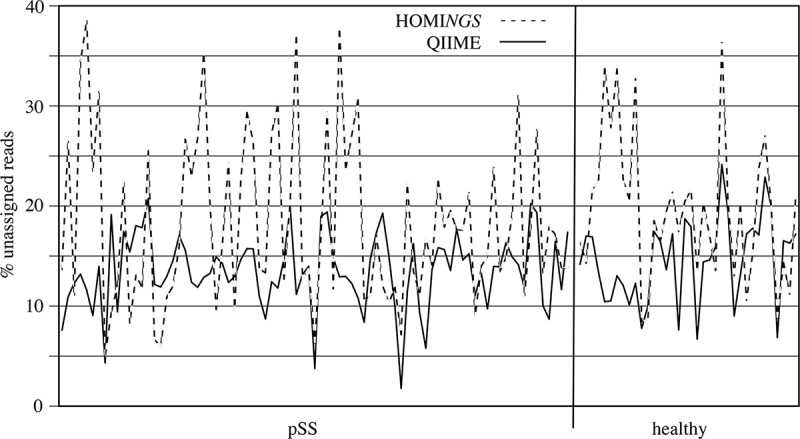
Sample-dependent percentage of unassigned reads in QIIME, and of unmatched reads in HOMI*NGS*, for all 119 samples. The vertical line separates pSS subjects (left) from healthy subjects (right).

Characteristics of the read datasets are shown in Table 2. The absolute number of reads after quality filtering varied between samples and was consistent between the two datasets. Small variations in total reads relate to the ProbeSeq chimera-removal algorithm. For unassigned reads, the absolute number and the percentage were always greater for HOMI*NGS*, which might be expected with a probe-based approach. However, Figure 2 shows good correspondence between sample-dependent differences for each dataset. In the HOMI*NGS* analysis, assignments at the species level typically exceeded those at the genus level (data not shown); 16 of 83 samples from pSS subjects and three of 36 samples from healthy subjects did not fit this pattern.

**Table 2.** Cumulative read characteristics for 119 MiSeq libraries analyzed by HOMI*NGS* and QIIME. na = not applicable.

	HOMI*NGS*		QIIME	
	Mean	Range	Mean	Range
Total reads	68,820	27,446–324,045	67,189	23,808–324,189
Assigned to 1 species probe	34,980	11,783–152,128	na	na
Assigned to 1 genus probe	21,215	3307–127,359	na	na
Total assigned reads	57,219	21,879–279,487	62,437	22,225–300,382
Unassigned reads	12,625	3697–68,917	4752	925–23,807
% assigned to 1 species probe	51.4	19.6–84.1	na	na
% assigned to 1 genus probe	29.9	9.6–75.4	na	na
Total % assigned	81.3	61.5–95.0	92.3	74.4–99.2
% unassigned	18.7	5.0–38.5	7.7	0.8–25.6

### Comparison of taxonomic assignments for plaque samples

Taxonomic assignment by QIIME identified 475 taxa across all samples. In HOMI*NGS*, 534 taxa have species-level probes and 348 were matched. Of the 117 genus-level probes, 77 were matched (Supplementary Table 2). Taxa corresponding to 20 unmatched species-level HOMI*NGS* probes were identified by QIIME. Of these, only *Actinomyces* sp. HMT 180 and TM7 [G-1] HMT 488 occurred in more than 10 samples at abundances >0.1% (data not shown). Five of the 20 were seen only in a single sample and at abundances <0.1% (data not shown).

**Figure 3.**
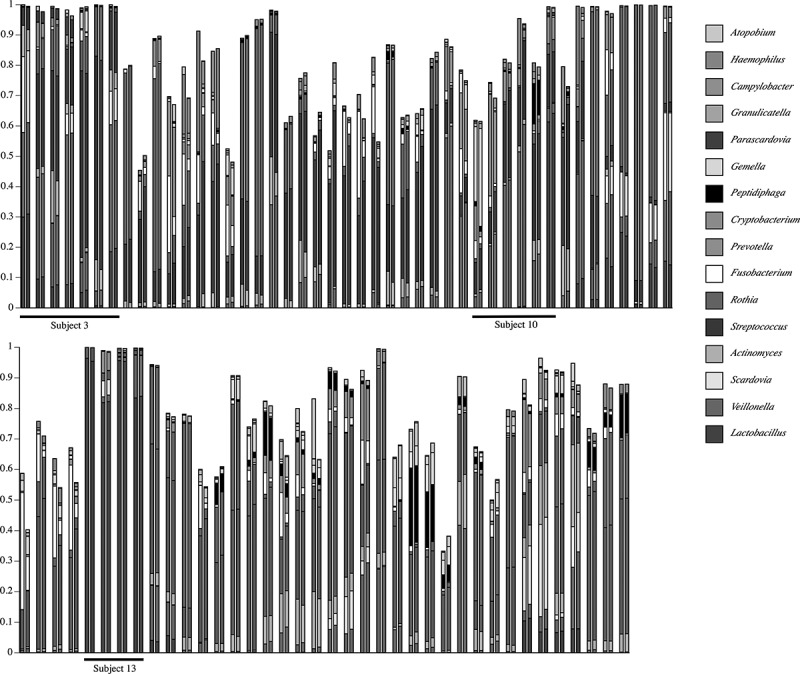
Map of genus-level taxonomic assignments in samples from pSS subjects. Only the 16 most-abundant genera in pSS Subject 3 (underlined samples at upper left) are shown. Each plaque sample is represented as a pair of columns. Taxonomic assignments by QIIME are shown in the left column and those by HOMI*NGS* in the right column. The underlined samples are from subjects with active caries. The seven samples from Subject 3 are examined in greater detail in subsequent figures.

**Figure 4.**
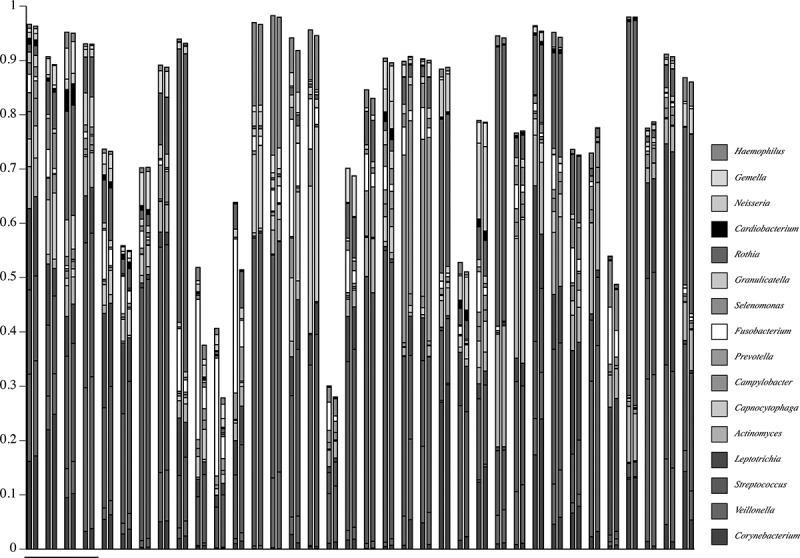
Map of genus-level taxonomic assignments in samples from healthy subjects. Only the 16 most-abundant genera for healthy Subject 8 (underlined samples) are shown. Each plaque sample is represented as a pair of columns in which taxonomic assignments by QIIME are shown in the left column and those by HOMI*NGS* in the right column. The samples from Subject 8 are examined in greater detail in subsequent figures.

A gross comparison (interpretable as a heat map) of community composition at the genus-level for all plaque samples from pSS subjects is shown in Figure 3, and for healthy subjects in Figure 4. The initial underlined columns in each chart show the 16 most abundant genera within that set of samples from a single subject: seven samples from Subject 3 in the pSS chart and four samples from Subject 8 in the healthy chart. This same set of genera is then used for the other samples. Thus, differential abundance of these particular genera generates sample-dependent differences in community coverage, *i.e*. relatively low coverage is seen in some samples. However, congruence between the two analysis methods is clear for most samples. The underlined samples in Figure 3 are from the three subjects with active caries. It is noteworthy that all four samples of pSS Subject 13 were taken from active caries lesions; *Lactobacillus* spp. made up at least 80% of these communities.

**Figure 5.**
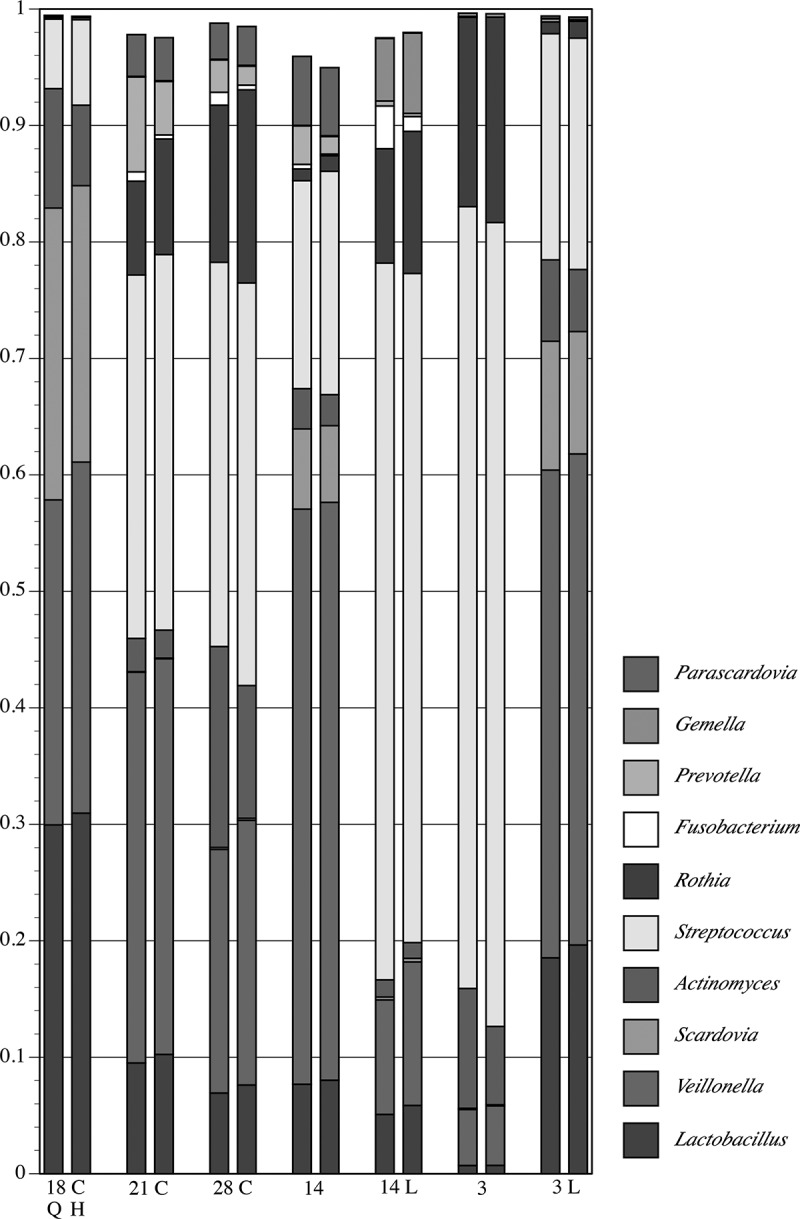
Detail of proportional genus abundance in the seven samples from pSS Subject 3 (the underlined samples at upper left in Figure 3). Paired comparisons of assignments by QIIME (Q, left column) with those by HOMI*NGS* (H, right column) for each tooth sample (tooth number, C = caries lesion, L = lingual surface).

**Figure 6.**
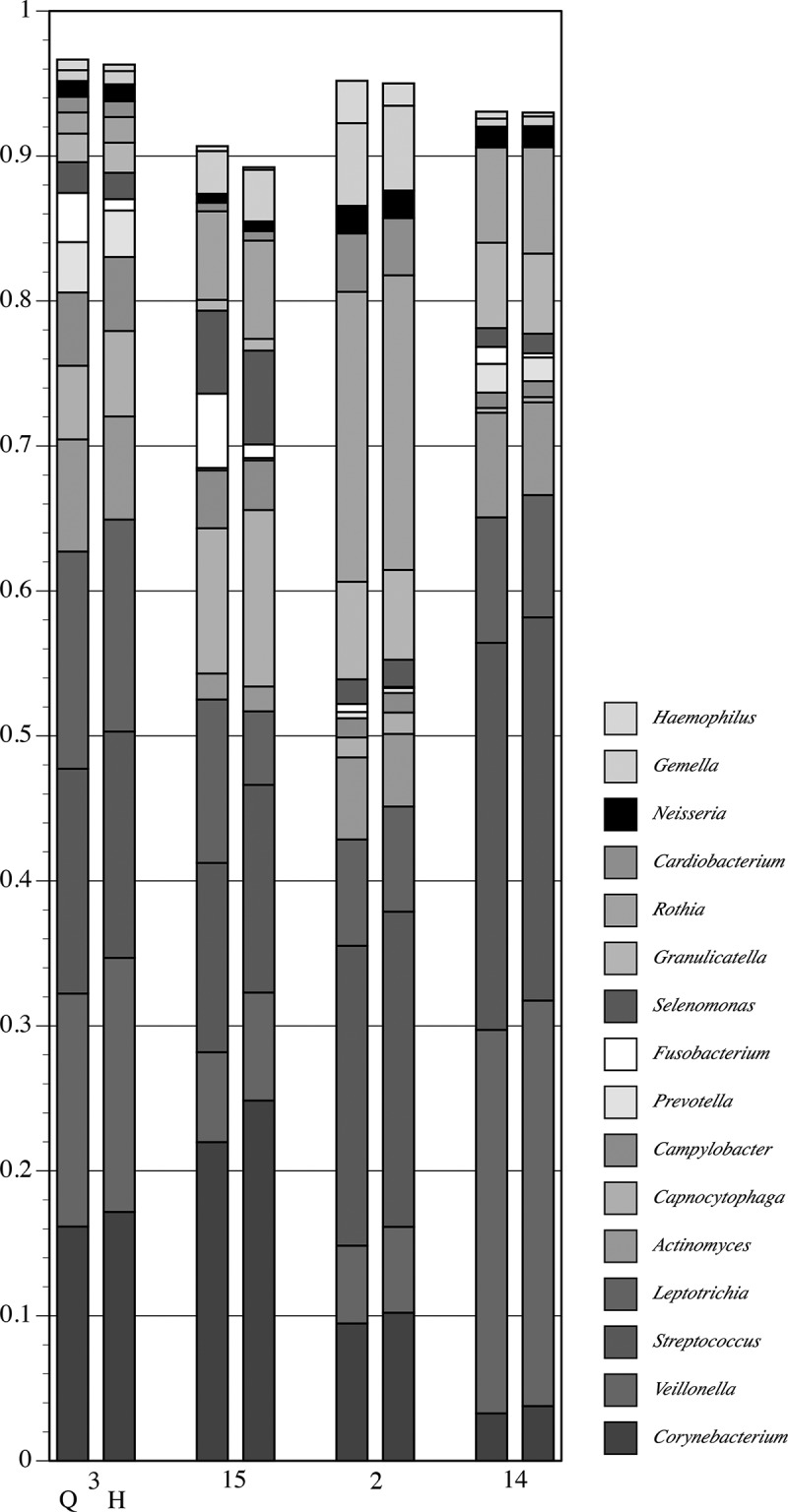
Detail of proportional genus abundance in healthy Subject 8 (the underlined samples in Figure 4). Paired comparison of assignments by QIIME (Q, left column) and by HOMI*NGS* (H, right column) for each tooth sample (tooth number).

Figure 5 shows a detailed comparison of the 10 most abundant genera in the seven samples from pSS Subject 3 (had active caries – samples from lesions are labeled C). These genera make up at least 95% of the community. As could be expected for a caries-active subject, *Lactobacillus* and *Veillonella* were prominent in all but one sample (3), and congruence between analysis methods is clear. Congruence for *Streptococcus* is likewise clear. The caries-associated genera *Scardovia* and *Parascardovia* occur in several samples and correlate well between analysis approaches. Congruence between methods for *Fusobacterium, Prevotella*, and *Actinomyces* is not as good as for the other genera, however, the overall community composition at the genus level is strikingly similar regardless of analysis approach. Figure 6 shows a comparison of the top 10 genera in the four samples from healthy Subject 8 (highlighted in Figure 4). While these genera comprise a large percentage of the community, the coverage overall is less than for the pSS community; one sample has only 90% coverage. This is predicted from prior knowledge of species diversity in plaque from healthy sites relative to that of sites tied to microbially influenced oral diseases. Similar to the pSS samples, the congruence between analysis approaches is striking, especially for the genera that make up the bulk of the community: *Corynebacterium, Veillonella, Streptococcus*, and *Rothia*. As was also true for the pSS samples in Figure 5, congruence between analysis methods for *Fusobacterium* is lower than for other genera.

**Figure 7.**
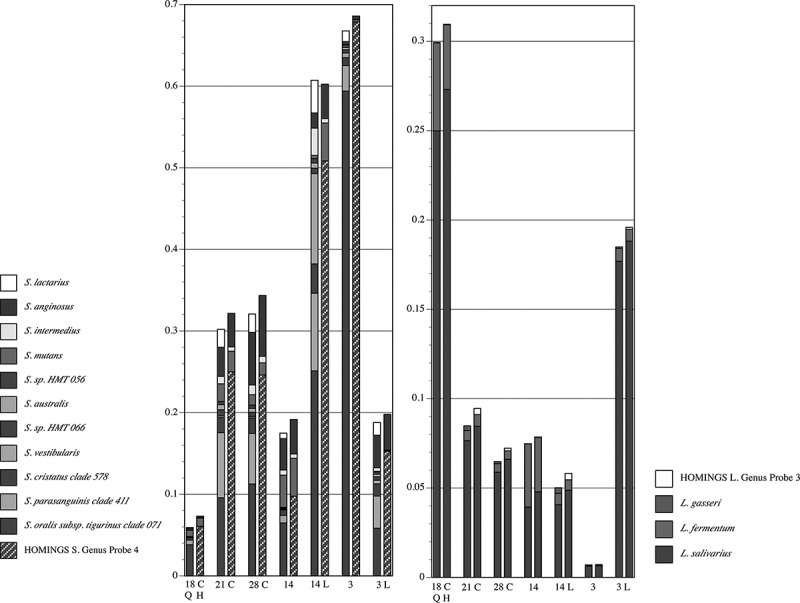
Proportional abundance of *Streptococcus* spp. and *Lactobacillus* spp. for pSS Subject 3 (samples in Figure 5). Paired comparisons of assignments by QIIME (Q, left columns) with those by HOMI*NGS* (H, right columns) for each tooth sample (tooth number, C = caries lesion, L = lingual surface). All species with abundance ≥0.005 (0.5%) in any sample are shown. For species that lack a species-level HOMI*NGS* probe, the corresponding genus-level probe is shown. HOMI*NGS Streptococcus* Genus Probe 4 (hatched blue block in the HOMI*NGS* columns) covers the species indicated by dark/light blue blocks in the QIIME columns.

Species-level comparisons within two major caries-related genera for pSS Subject 3 are presented in Figure 7. A disadvantage of HOMI*NGS* is that it does not distinguish between many commensal streptococci; instead, a genus-level probe is necessary. The left panel in Figure 9 demonstrates that the summed abundances of commensal streptococci identified in QIIME relate well to the abundance of HOMI*NGS* Genus Probe 4 which covers those species. Two commensal streptococci were identified by QIIME (*S. anginosus* and *S. intermedius*) that are represented by species-level HOMI*NGS* probes and, with the exception of one sample (14 L), congruence between methods was seen for each. Importantly, a species-level probe exists for the pathogen *S. mutans*, and congruence between QIIME and HOMI*NGS* is also clear for this species except in the same sample in which *S. anginosus* and *S. intermedius* were not well correlated. Interestingly, QIIME identified moderate amounts of *S. lactarius*, an organism originally isolated from breast milk. For clarity, a handful of QIIME-identified streptococci and HOMI*NGS* streptococcal probe matches that occurred at levels <0.5% are not shown in Figure 9. QIIME identified nine additional streptococci at these low levels. Of these, six can be accounted for by HOMI*NGS* Genus Probe 4, and one (*S. gordonii*) is accounted for by HOMI*NGS* Genus Probe 1 which was likewise matched at <0.5%. Thus, HOMI*NGS* did not account for two species identified at low levels by QIIME: *S. constellatus*, and *S. thermophilus*. Three HOMI*NGS* probes were matched below the 0.5% threshold. As noted above, HOMI*NGS* Genus Probe 1 correlated with *S. gordonii* which was also identified at levels below 0.5% by QIIME, but *S. sanguinis* was not identified by QIIME, nor were the two common species covered by the low-level match to HOMI*NGS* Genus Probe 3 (*S. salivarius* and *S. vestibularis*).

Of importance to caries, the three major *Lactobacillus* species also showed congruence (right panel). As for the streptococci, a HOMI*NGS* genus-level probe correlates well with one of its corresponding species identified by QIIME: Genus Probe 3 with *L.gasseri*. As occurred with the streptococci, some *Lactobacillus* species were detected at levels <0.5% by each method. QIIME identified seven, of which only *L. ultenensis* could not be accounted for by HOMI*NGS*. Of the four HOMI*NGS* probes matched below 0.5%, all have corresponding species identified at low levels by QIIME.

**Figure 8.**
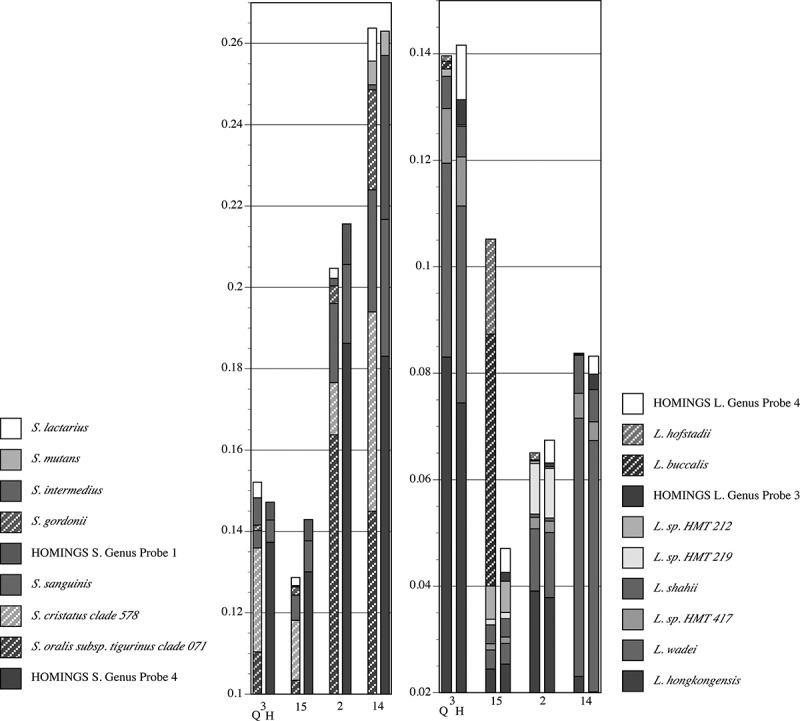
Proportional abundance of *Streptococcus* spp. and *Leptotrichia* spp. for healthy Subject 8 (samples in Figure 6). Paired comparisons of assignments by QIIME (Q, left columns) with those by HOMI*NGS* (H, right columns) for each tooth sample (tooth number). All species with abundances greater than 0.005 (0.5%) in any sample are shown. For species that lack a HOMI*NGS* probe, the corresponding genus-level probe is shown. HOMI*NGS Streptococcus* Genus Probe 4 (hatched blue block in the HOMI*NGS* columns) covers the species indicated by dark/light blue blocks in the QIIME columns. HOMI*NGS Streptococcus* Genus Probe 1 covers *S. gordonii*. HOMI*NGS Leptotrichia* Genus Probe 3 (brown) corresponds to *L. hofstadiii* and *L. buccalis* (brown hatched bars) in the QIIME analysis. HOMI*NGS* Genus Probe 4 (white) had no equivalent species in the QIIME analysis.

Species-level composition for streptococci and *Leptotrichia* spp. from healthy Subject 8 is shown in Figure 8. Congruence between QIIME-identified commensal streptococci and HOMI*NGS* Genus Probe 4 is high in three of the four samples; divergence occurs in sample 14. The correlation between *S. gordonii* identified by QIIME and its corresponding HOMI*NGS* Genus Probe 1 follows the same pattern. Importantly, congruence for *S. mutans* is clear. However, *S. intermedius* and *S. lactarius* are identified only by QIIME. Not appearing in the figure (<0.5% of the community) are 11 species identified by QIIME. All but two (*S. anginosus* and *S. vestibularis*) are covered by Genus Probe 4. Three HOMI*NGS* probes were matched at <0.5% and all have corresponding QIIME low-level matches. Thus, for streptococci in these particular samples, the analysis approaches differ only with respect to *S. anginosus* and *S. vestibularis*.

For *Leptotrichia* spp., congruence between the two approaches is again striking. One major discrepancy occurs: the proportion of *L. hofstadii* and *L. buccalis* in sample 15 as detected by QIIME was nearly 20 times greater than was the proportion of the corresponding HOMI*NGS* Genus Probe 3. The lack of correspondence occurs in the other samples as well, but the degree of discrepancy is closer to two-fold, and the more sensitive approach varies between the samples. Another discrepancy is the small amounts of HOMI*NGS* Genus Probe 4, for which no covered species occur in the QIIME analysis. Six species were present at levels below <0.5% in the QIIME analysis (*i.e*. do not appear in the figure), and three of these were also found in the HOMI*NGS* low-level matches. Two additional HOMI*NGS* probes matched at <0.5% were not found in by QIIME. Thus, the proportion of five *Leptotrichia* spp. present at low levels was not congruent between approaches.

**Figure 9.**
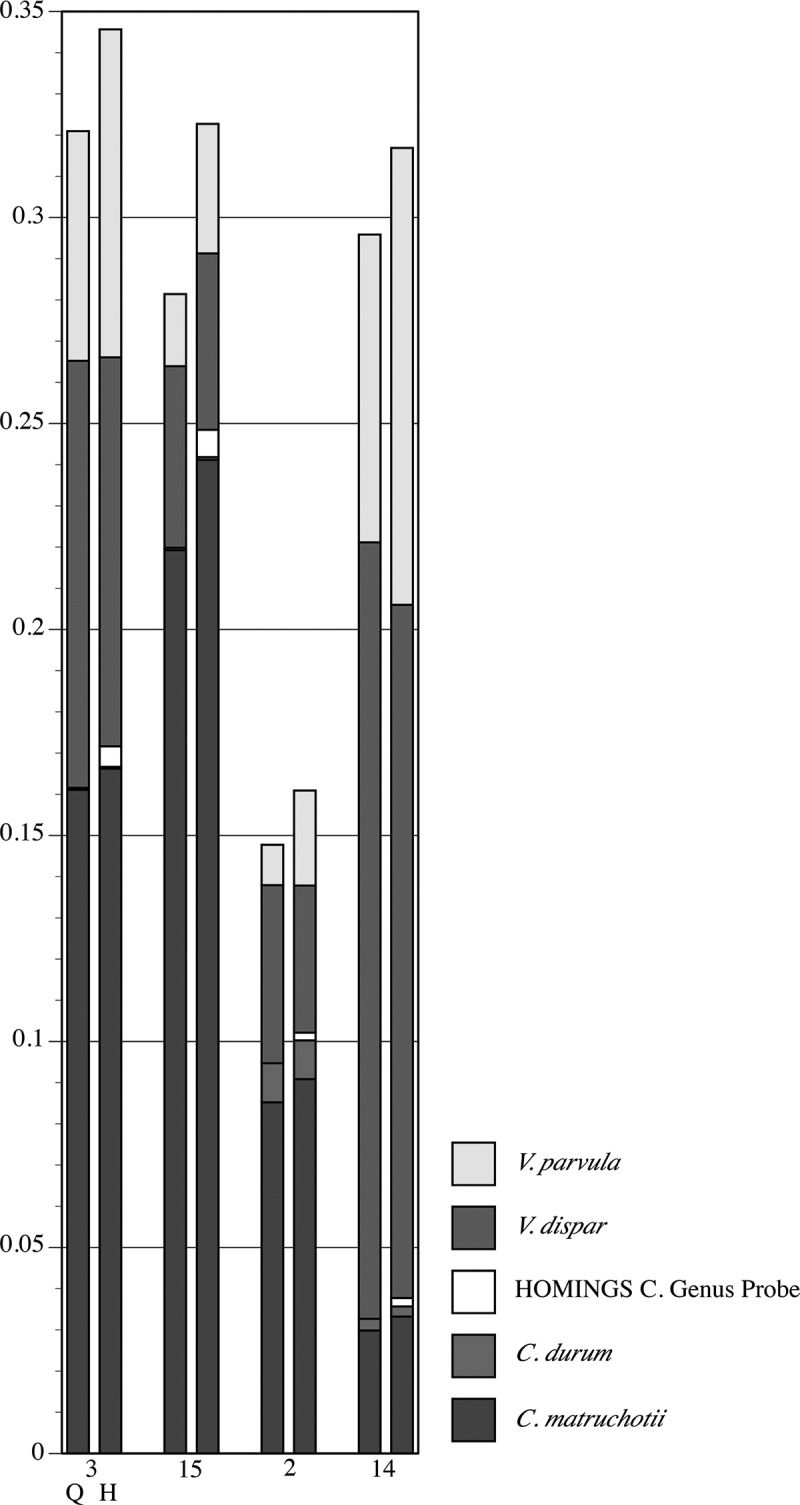
Proportional abundance of *Corynebacterium* spp. and *Veillonella* spp. in healthy Subject 8 (samples in Figure 6). Paired comparisons of QIIME (left bar) with HOMI*NGS* (right bar) for each tooth sample (tooth number). All species with abundances greater than 0.005 (0.5%) in any sample are shown. The HOMI*NGS Corynebacterium* Genus Probe covers *C. durum* and *C. matruchiotti.*

Figure 9 shows the proportions of *Veillonella* spp. and *Corynebacterium* spp. as detected by QIIME and HOM*INGS*. Congruence is clear, with the only discrepancy being the small amounts of HOMI*NGS* Genus Probe for *Corynbacterium* spp.. Coverage by this probe includes many species, and overlaps with *C. durum* and *C. matruchotii*. Aside from *durum* and *matruchotii*, none of species covered by the Genus Probe were identified by QIIME. Therefore, this is either a situation in which some sequences representative of *C. durum* and *C. matruchotii* were not removed in the initial match to species probes, or a case in which QIIME failed to correctly identify *Corynebacterium* spp. that would match with the HOMI*NGS* genus-level probe. Detection of common *Veillonella* spp. (*atypcia, denticariosa, rogosae*) by QIIME occurred at levels <0.5% in three of the samples (*i.e*. do not appear in the graph); HOMI*NGS* Genus Probe 2 corresponds with these and was detected at low levels.

Overall, both analysis methods show the composition of plaque communities from healthy as well as from pSS subjects, including the presence of caries pathogens and the recognition of inter-individual variability, to be consistent with that described in the literature [3,28–30]. Differences in community composition as determined by the two methods are few and generally of limited magnitude.

### Comparison of diversity by QIIME

**Figure 10.**
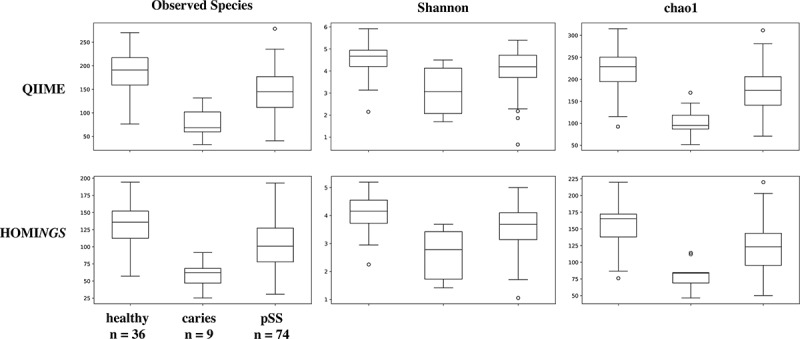
Alpha diversity comparing samples from healthy subjects, caries samples from pSS subjects, and samples from sound tooth surfaces of pSS subjects.

**Figure 11.**
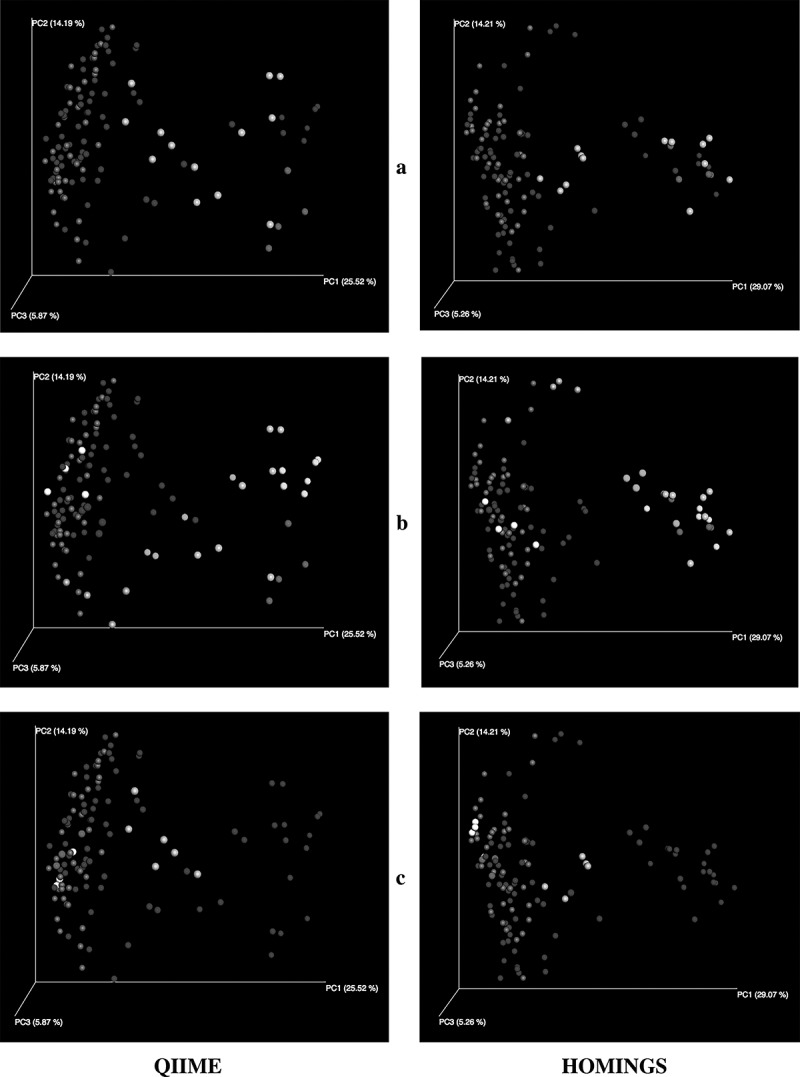
PCoA plots of unweighted UniFrac distances. (a) Samples from healthy subjects in dark green. Samples from pSS subjects without active caries in dark blue. Samples from the three pSS subjects with active caries in bright green, red and orange. (b) Samples from healthy subjects in green. Samples from pSS subjects with measurable salivary flow in blue. Six pSS subjects had no measurable salivary flow. Samples from five of those subjects (yellow, light blue, bright green, orange, red) are separated from those of healthy subjects and other pSS subjects. Samples in red and bright green are from pSS subjects with caries who also had no measurable flow (see panel a). Samples from the sixth subject (white) are within the cluster of healthy samples. (c) Samples from healthy subjects in green. Samples from pSS subjects with reduced or no measurable salivary flow in blue. Three pSS subjects had normal salivary flow. Samples from two of those subjects (red, white) are within the cluster of healthy samples. The third subject (orange) had active caries (see panel a).

Alpha diversity measurements distinguish the three subject cohorts shown in Figure 10. The measures for plaque from sound tooth surfaces in pSS subjects are close to those for plaque from teeth of pSS subjects, while samples from carious sites are clear outliers. The only difference related to taxonomic assignment method for each diversity measurement is in absolute value. Beta diversity expressed as PCoA plots of Unifrac distances allows comparison of community composition at the level of individual samples as well as across individual subjects. While weighted UniFrac analysis showed some separation between various groups of subjects (data not shown), differences were clearest in the unweighted analysis (Figure 11), and the plots show close correlation between the two analyses. Samples from healthy subjects formed a cluster; those from diseased subjects overlapped that cluster but roughly one-third had greater values for PC1 and, as expected from existing knowledge of caries communities, most samples from subjects with caries were clearly separated from those of healthy subjects (Figure 11(a)). Salivary flow was likewise related to differences in PC1; samples from five of the six pSS subjects with no measurable flow were separated from those of healthy subjects (Figure 11(b)). Although reduced salivary flow is a typical feature of pSS, it is not a requirement for the diagnosis. In the present subject cohort, two pSS subjects had flow rates in the normal range, and samples from those subjects were located within the cluster of samples from healthy subjects (Figure 11(c)), especially with respect to PC1.

#### LEfSe analysis

**Figure 12.**
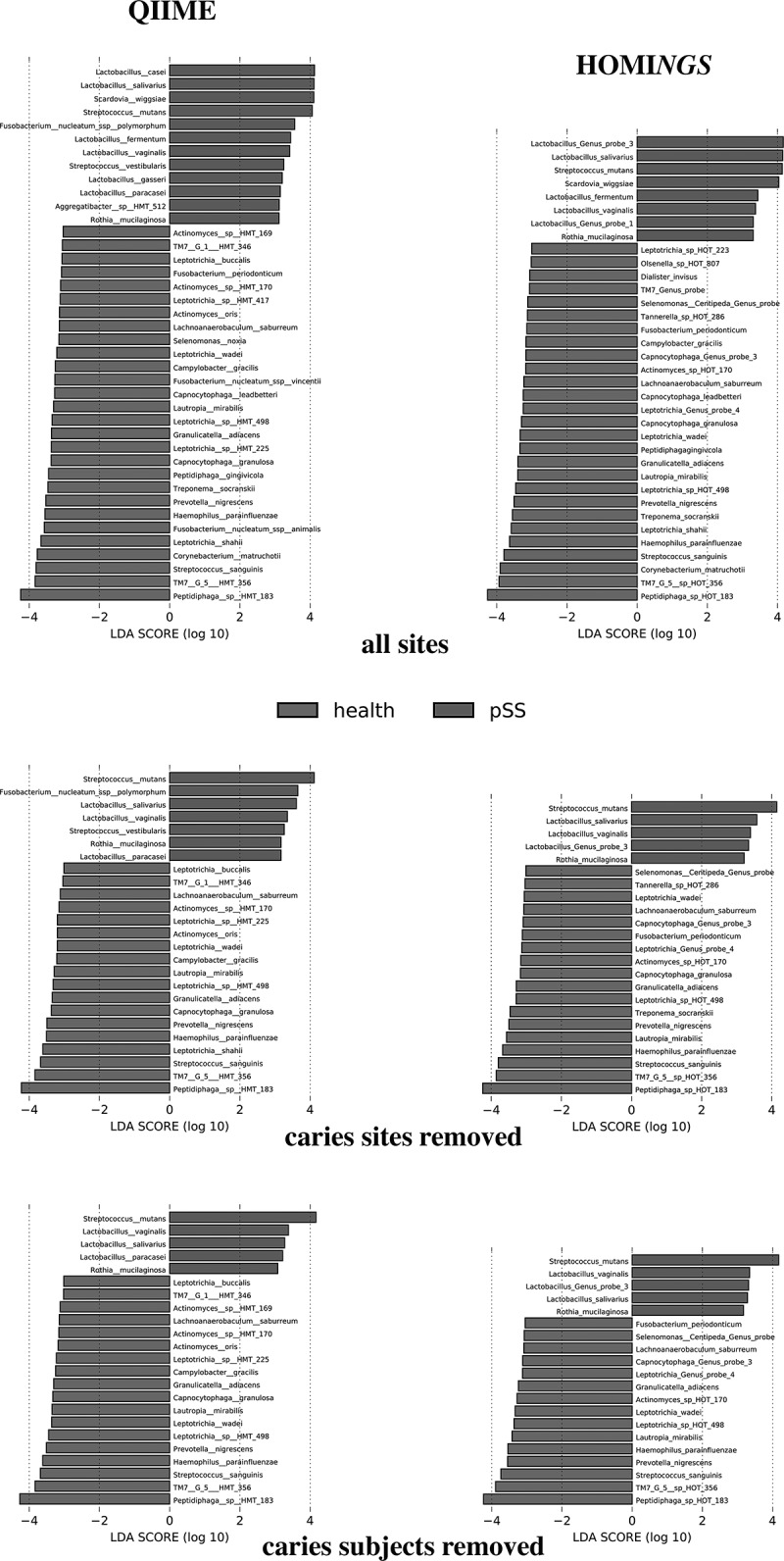
LEfSe analysis of abundance values for QIIME species ≥0.01% in any sample (left column) and HOMI*NGS* probes (genus- and species-level) ≥0.01% of total probe counts in any sample (right column). All sites (top row), when samples from caries lesions are removed (middle row), and when samples from all subjects with caries are removed (bottom row). HMT refers to designations for unnamed/uncultured species in the eHOMD.

Discriminatory species within biofilm communities of pSS subjects and healthy subjects were sought using LDA Effect Size (LEfSe) [27]. Figure 12 presents results for all sites (top row of panels), when samples from caries lesions were removed (center row), and when all samples from subjects with active caries were removed (bottom row). Regardless of analysis method, lactobacilli and Scardovia *wiggsiae* were over-represented in pSS communities when all sites were used. *Lactobacillus* spp. were also discriminators. *L. salivarius, L. vaginalis* and *L. fermentum* were present in both methods. HOMI*NGS* Lactobacillus Genus Probe 3 was over-represented in pSS subjects, and the species covered by this probe (*casei, paracasei* and *gasseri*) were all included in the QIIME results. *Rothia mucilaginosa*, commonly thought of as a component of healthy plaque, is also prominent in pSS subjects regardless of analysis method. Analysis of the QIIME dataset yielded *Fusobacterium nucleatum, Aggregatibacter* HMT 512 and *Streptococcus vestibularis* as discriminators – these were not identified using the HOMI*NGS* dataset. Discriminators for communities from healthy subjects were nearly identical regardless of analysis method; differences between QIIME taxonomy and species-level HOMI*NGS* probe matches were primarily in relative magnitude (position top-to-bottom of the red bars) rather than the presence of any single organism. An exception was identification of three *Fusobacterium* spp. using the QIIME dataset – only *F. periodonticum* was identified using the HOMI*NGS* dataset, but it was at the same level of significance as in the QIIME results. Several organisms commonly associated with healthy supragingival biofilms were prominent: *Streptococcus sanguinis, Corynebacterium matruchotii, Haemophilus parainfluenzae* and *Granulicatella adiacens*. Of note, commensal streptococci, in general, were not discriminatory, and certain taxa associated with subgingival communities (*Prevotella nigrescens, Treponema socranskii* and *Selenomonas noxia*) were over-represented. Also, interesting is identification of *Peptidiphaga* spp. as discriminators for healthy subjects. These sets of discriminating species for pSS and health remained constant as caries-associated samples were removed from the pSS data, *i.e*. when comparing the panels top-to-bottom. Only *Scardovia wiggsiae* disappeared when caries lesion samples were removed.

## Discussion

Parallel analysis of 16S PCR product mixtures (Figure 1) shows that QIIME and ProbeSeq deliver nearly identical results; in only 1 of the 10 mixtures did results differ, and the difference was in a single product. However, these contrived samples are not representative of the variation generated when a natural community is taken through the complete analysis pipeline, *i.e*. DNA extraction, 16S PCR amplification, and sequence library generation. It is therefore critical to examine natural samples, as was done here through parallel analysis of 119 MiSeq libraries generated from dental plaque. Equivalence of basic numerical characteristics across the sequence datasets would be desirable at the outset, and the two datasets are indeed very much alike (Table 2). Relative community composition at the genus level across the 119 plaque samples was highly similar between the tree-based and the probe-based approach (Figures 3 and 4). This suggests not only that differences in database coverage between the two methods are not a major factor at the genus level, but also that coverage of the eHOMD database by the inherently restricted HOMI*NGS* probe set is inclusive with respect to the prominent oral genera. These points are further reinforced by a detailed comparison at the genus level within sets of samples from individual subjects (Figures 5 and 6). As has been understood for some time, the proportions of the major oral organisms vary somewhat across individuals and greatly between health and disease. The present data likewise reinforce these traits at the level of the entire dataset (Figure 3*vs*. Figure 4) and at the level of single individuals (Figure 5*vs*. Figure 6).

While equivalency between the analysis approaches at the genus level is critical, the major variation between individuals, and between health and disease, should be most clearly apparent at the species level. Therefore, species-level taxonomy is necessary to fully assess the importance of differences in composition. One inherent disadvantage of the HOMI*NGS* method is its inability to discriminate commensal streptococci; most of commensal streptococci are covered only by genus-level probes. The striking correlation of, *e.g*. streptococcal genus-level probe abundances with the QIIME abundances of the species covered by those probes, speaks to the accuracy and sensitivity of the otherwise limited HOMI*NGS* taxonomic assignment (Figures 7, 8, and 9). In only one case was a major discrepancy seen, and it was unrelated to particular samples (*Leptotrichia* Genus Probe 3, Figure 8). The pSS samples used here, especially those sampled directly from caries lesions, provide an opportunity to assess correlation between methods with respect to caries pathogens. A species-specific probe exists for *Streptococcus mutans* as well as for the two most prominent *Lactobacillus* spp. in the samples from pSS Subject 3. The congruence between analysis methods for these species was impressive (Figure 7). Thus, at least for this particular set of samples, the major caries pathogens are covered well by both methods.

Comparison of alpha diversity for three subsamples (healthy teeth in healthy subjects, healthy teeth in pSS subjects, and caries lesions in pSS subjects) shows little influence of analysis method – for each diversity criterion, the range within each sample group is nearly identical between the methods. However, the absolute values for the HOMI*NGS* approach were slightly lower than for QIIME. Thus, a more rigorous view of diversity than is possible from simple comparisons of relative abundance reveals a slight difference related most likely to database coverage. Relative to the QIIME taxonomy, the HOMI*NGS* approach seems to show inherently reduced depth and lower variation which may be associated with the fixed number of probes and the grouping together of species at the genus level for numerous taxa (genus-level probes). Nonetheless, the effect of this difference on beta diversity was very limited. PCoA plots show the data points for individual samples, as well as the subject-dependent relationship between those points, to be independent of analysis approach. Larger differences in magnitude along PC2 and 3 were seen for the QIIME taxonomy than for the HOMI*NGS* data, but the two datasets were similar with respect to PC1. Together, the diversity analyses suggest that the datasets are largely equivalent. It is important to recognize that a sufficient number of appropriate healthy control group subjects (older females with normal salivary flow) have not yet been enrolled through our protocols, thus the clinical significance of these results for microbial ecology in pSS is not clear. However, the present healthy control group (young individuals of low caries experience, 60% male) may represent a greater divergence from the Sjögren’s group than would an age- and sex-matched cohort. Thus, alpha and beta diversity analysis of HOMI*NGS* and of tree-based data support expected differences between these cohorts related to salivary flow and active caries, and they support use of both datasets in for LEfSe analysis.

Classical bacteriological analyses, as well as molecular taxonomic studies, of plaque and of saliva from subjects with salivary hypofunction show differences in overall community composition compared to that from healthy subjects, particularly with respect to caries pathogens such as *Streptococcus mutans* and lactobacilli [3,31–33]. QIIME analysis of alpha and beta diversity in the present datasets likewise show differences in community composition between the healthy and pSS subjects, and those results support subsequent use of LEfSe as an independent statistical measure of taxa that could discriminate healthy from pSS subjects. As expected for the healthy subject population, several species associated with health are over-represented. Unexpectedly, commensal streptococci, in general, did not belong to the over-represented species; rather, certain species associated with the subgingival environment were over-represented. These observations remained constant when caries-associated samples were removed. As would be expected for the pSS subjects, *S. mutans* and lactobacilli were identified as discriminating factors, and they likewise remained constant when data from caries-active subjects were excluded, but with one interesting exception – the disappearance of *Scardovia wiggsiae* when caries lesion samples are removed. Together, the LEfSe data suggest that, in addition to the caries pathogens already known to be associated with pSS, bacteria associated with the gingival crevice could be important in the microbial ecology of the disease [3]. As noted previously, clinical significance of the present results is clearly limited because a relevant control group (older women with normal salivary flow but having caries experience similar to that of the pSS subjects) has yet to be sampled in sufficient numbers within our protocols. In addition, the number of caries lesions in the datasets is small. However, a reasonable interpretation of the present data is that taxa associated with the subgingival environment might be important, and that this observation should be investigated further with a clinically relevant control group. Likewise, it should be noted that certain taxa can be overrepresented in a given sample simply because of the 16S region selected for amplification [34,35]. The V1-V2 region is recognized as the most information rich and has been used for oral microbiome analysis [15]. Future studies to address the role of subgingival bacteria in pSS might benefit from application of a different primer set. Despite the inherent bias associated with primer selection, the datasets produced by the two analysis methods in this study are internally consistent; assessment of analysis equivalency as well as interpretation of the comparative aspects (diversity/LEfSe) are not affected.

The present study shows that analysis of identical V3-V4 MiSeq libraries using the HOMI*NGS* probe-based approach and the QIIME implementation of a tree-based approach yield highly congruent results. The known limitation of HOMI*NGS* in identification of commensal streptococci is clearly demonstrated here but, at the same time, species-specific and genus-specific probe accuracy have been validated using natural samples. Thus, the present study strengthens integrity and conclusive power of the probe-based HOMI*NGS* approach.
